# Single-atom materials boosting wearable orthogonal uric acid detection

**DOI:** 10.1007/s44258-024-00027-1

**Published:** 2024-09-20

**Authors:** Shichao Ding, Sitao Li, Zhaoyuan Lyu, Jiachi Zhou, Selene Tang, Lingzhe Fang, Wenjie Zang, Pengtao Zhang, Sunjae Kim, Tao Li, Xiaoqing Pan, Dan Du, Yuehe Lin, Joseph Wang

**Affiliations:** 1 Aiiso Yufeng Li Family Department of Chemical and Nano Engineering, University of California San Diego, La Jolla, CA 92093, USA; 2 Shu Chien-Gene Lay Department of Bioengineering, University of California San Diego, La Jolla, CA 92093, USA; 3 School of Mechanical and Materials Engineering, Washington State University, PO Box 642920, Pullman, WA 99164, USA; 4 Department of Chemistry and Biochemistry, Northern Illinois University, DeKalb, IL 60115, USA; 5 Department of Physics and Astronomy, Department of Materials Science and Engineering, Irvine Materials Research Institute (IMRI), University of California Irvine, Irvine, CA 92697, USA; 6 X-Ray Science Division, Argonne National Laboratory, Lemont, IL 60439, USA

**Keywords:** Wearable sensor, Single-atom materials, Sweat sensing, Orthogonal detection, Uric acid

## Abstract

Uric acid (UA) is a vital biomarker for the diagnosis and management of various health conditions, including cardiovascular diseases, gout, kidney disorders, metabolic syndrome, and wound healing. Despite significant advances in wearable sensor technology, challenges persist in developing wearable sensors that are capable of maintaining high sensitivity, selectivity, and stability. In this study, we present an epidermal sensing platform enhanced with single-atom materials (SAMs) designed for flexible and orthogonal electrochemical detection of UA. We designed and synthesized an SAM with Fe-N_5_ active sites to boost the electrochemical sensing signals, integrating it with laser-engraved graphene (LEG) to fabricate a wearable SAM-based UA patch sensor. This design provides superior UA detection performance compared to sensors based on conventional nanomaterials. In addition, we enhanced the detection accuracy and range by using an orthogonal approach that combines direct oxidation through differential pulse voltammetry (DPV) along with parallel biocatalytic amperometric detection. The resulting SAM-based UA orthogonal sensor patch demonstrated exceptional performance in wearable applications through tests measuring sweat UA levels in subjects before and after consuming a purine-rich diet.

## Graphical Abstract

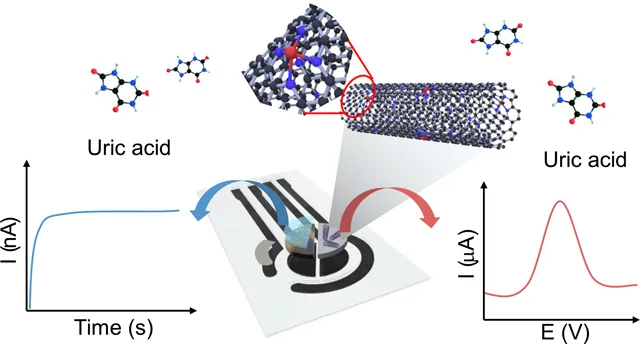

## Introduction

Wearable devices have recently demonstrated considerable promise for advancing human healthcare [1–5]. These devices are designed to be worn on the body and facilitate continuous, real-time tracking of various physiological parameters, such as heart rate and body temperature, as well as biomarkers like glucose, lactate, and uric acid (UA) [6–13]. Flexible sensors, as core components of wearable devices, have promising applications in health status monitoring and electronic skin [14–17]. Despite extensive research efforts towards developing wearable sensors, challenges remain in integrating these sensors into a wearable platform while maintaining high detection sensitivity, selectivity, and stability [18].

Designing novel active materials for sensing interfaces is crucial for enhancing the detection performance of biosensors [19, 20]. For instance, Li et al. reported that a flexible graphene electrode decorated with Fe_3_O_4_ nanoparticles exhibits enhanced selectivity and sensitivity for the electrochemical sensing of different biomarkers (like dopamine and serotonin) compared to a bare graphene sensor [21]. To further enhance the performance of active sensing materials, advanced synthesis techniques downsize their active site size from the nano level to the atomic level [22, 23]. These materials, typically called single-atom materials (SAMs), are characterized by their exclusively isolated active metal sites [24, 25]. SAMs have garnered significant attention owing to their unique activity and have been incorporated into various signal-amplified electrochemical sensing and biosensing applications [26–29]. The advantages of SAMs include maximizing the utilization of active metals to enhance sensitivity while simultaneously mitigating issues of low sensing selectivity caused by the inhomogeneous distribution of materials [30]. To illustrate, our group demonstrated that an electrochemical sensor based on a Fe–N-C SAM-modified electrode exhibits excellent sensitivity for hydrogen peroxide (H_2_O_2_) detection [31]. Li et al. developed p-block In_1_–N–C SAM that exhibits a low energy barrier in the catalytic oxidations of dopamine and the ability to be assembled into an electrochemical sensor for highly sensitive dopamine monitoring [32]. Given these advantages, we believe there is tremendous potential in using SAMs to boost the performance of flexible and wearable sensors for healthcare applications involving diverse on-body monitoring [33].

In this work, we present a SAM-enhanced epidermal sensing platform that offers excellent flexibility for the orthogonal electrochemical detection of UA (Fig. 1a i). UA was selected as the targeted biomarker as its concentrations in different body fluids are essential for diagnosing, managing, and preventing various diseases, including cardiovascular health [34], gout [35], different kidney diseases [36], metabolic syndrome [37], wound conditions [38], and more [39]. Hence, current wearable technologies have been focusing primarily on UA detection in sweat [40]. In this work, a SAM with Fe-N_5_ active sites was designed (Fig. 1a ii) and assembled with laser-engraved graphene (LEG, Fig. 1b)-based sensors. To improve the detection sensitivity and expand the range of sensing concentration, an orthogonal detection method is introduced, integrating direct oxidation via differential pulse voltammetry (DPV) (at WE1) and an enzyme-cascade biocatalytic amperometric response (at WE 2) (Fig. 1c). We discovered that both wearable electrochemical detection schemes for UA are improved using single-atomic site materials compared to conventional nanomaterials. The outstanding performance and versatility in wearable applications of this new SAM-based UA patch sensor were demonstrated in monitoring sweat UA in different subjects after overnight fasting and subsequent consumption of a purine-rich diet.

## Experimental section

### Materials

All chemicals were used as received without further purification. Phosphate-buffered saline (PBS), uric acid (UA), D-(+)-glucose, uricase from Candida sp. (≥ 2 units/mg), bovine serum albumin (BSA), sodium chloride (NaCl), potassium chloride (KCl), lactic acid, tyrosine, toluene, ethanol, chitosan, acetic acid, H_2_O_2_, chitosan, sulfuric acid (H_2_SO_4_), polyimide film, and Nafion were obtained from Sigma-Aldrich (USA). Canned sardines were purchased from a local Costco Wholesale store. Styrene-ethylene butylene-styrene block copolymer (SEBS) MD1648 was supported by Kraton (Houston, TX, USA).

### Synthesis of FeN_5_ SAM

The synthesis process is described in the Supplementary Figure S2. CNT was treated under NH_3_ at 900 °C for 10 h to dope N atoms on the carbon matrix. Then, the 300 mg N enriched CNT was dissolved in 40 mL of DMF, and then mixed with 30 mg FePc. After 30 min ultrasound treatment and following 5 h of stirring, N-CNT@FePc was collected by centrifugation, washed several times with H_2_O, and dried under the vacuum condition at 60 °C for overnight. Finally, the N-CNT@FePc was prepared by a low-temperature calcining process under N_2_ at 300 °C for 1 h to achieve the FeN_5_ SAM sample.

### Fabrication of the wearable sensor

The LEGs were fabricated on a polyimide film using an xTool F1 laser engraver, with optimized parameters of power 60%, speed 3300 mm/s, 300 lines per cm, bi-directional engraving mode, and blue light laser type. A prepared SEBS solution (40 wt% in toluene) was then cast onto the obtained LEGs-PI film via a doctor blade and then peeled off from the PI film after overnight drying. Once the LEGs was successfully transferred to the SEBS film, working electrode 1 (WE 1) was fabricated by drop-casting a solution of 0.2 mg/mL FeN_5_ SAM in 0.05% Nafion in ethanol onto the LEGs in four 1 mL increments, totaling 4 mL. The electrode sensors were then immersed in 5% H_2_O_2_ in 0.5 M H_2_SO_4_ for 1 h. For working electrode 2 (WE 2), a solution of 3.2 mg BSA and 4.8 mg uricase in 320 mL PBS was mixed in 1:1 ratio with 1 wt% chitosan in 0.1 M acetic acid. Following the same procedure as for WE 1, 7 mL of the above solution was drop-casted onto each electrode, which was then incubated at −4 °C for 4 h.

### On-Body sweat sensing

On-body sweat UA sensing was performed on three healthy subjects, including one male and two females. After a 12-h overnight fast, the subjects underwent 10 ~ 20 min of intense exercise to induce sweating. Subjects 2 and 3 were then given a purine-rich diet (250 g of canned sardines), and subject 1 was given a high purine-rich diet (500 g of canned sardines). Data was collected every two hours during intense workouts.

## Result and discussion

An infrared laser engraver machine was used to fabricate the key circuits of the sensor (Supplementary Fig S1a). Specifically, the laser engraver was used to create graphene structures (Fig. 1b) [41] by scribing directly onto a polyimide substrate. The obtained LEG was then transferred to a flexible thermoelastic substrate (Supplementary Figures S1b–c) styrene–ethylene butylene–styrene block copolymer (SEBS), which is adhesive and biocompatible, thereby making it suitable for further wearable applications. A screen-printing technique was utilized to print the flexible AgCl ink onto one LEG electrode (Supplementary Fig S1d) to act as the reference electrode. The Fe-based SAM with dispersed Fe-N_5_ active metal sites was designed and synthesized to enhance the orthogonal UA detection performance. Herein, the FeN_5_ SAMs were synthesized by calcining iron (II) phthalocyanine (FePc) coupled with N-enriched carbon nanotubes (CNTs) at 300 °C for 1 h (details in the Experimental section). This low-temperature calcining process transforms the carbon-containing molecular complexes into solid anchor single-atom sites while preserving the coordination environments of the metal atoms [42].(Supplementary Figure S2) The assembled SAM-based sensor features two working electrodes (WEs) for orthogonal UA detection (Fig. 1c). WE 1 is fabricated by drop-casting SAM mixed Nafion solution onto the surface of LEG. A current peak from the non-enzymatic electrochemical DPV sensing corresponds to the oxidation reaction of UA, which involves the transfer of two-electron/two-proton. WE 2 is a SAM-enzyme cascade electrode. The biosensing principle involves immobilizing uricase, which catalyzes the conversion of UA into allatonin and H_2_O_2_. This H_2_O_2_ is further reduced to H_2_O, and its response is collected via chronoamperometry (CA). The final sensor, noted for its excellent flexibility, is shown in Fig. 1d.

X-ray absorption spectroscopy (XAS) analysis was conducted to determine the chemical state and coordination environment of the Fe active sites. As shown in Fig. 2a, the X-ray absorption near-edge structure (XANES) spectra indicate that the average valence state of Fe atoms in FeN_5_ SAM lies between Fe^2+^ and Fe^3+^ as the absorption edge position of FeN_5_ SAM lies between FeO and Fe_2_O_3_ [43]. The Fourier transforms of extended X-ray absorption fine structure (FT-EXAFS) exhibit a prominent peak at 1.52 Å (Fig. 2b), which is attributed to the Fe–N first coordination shell [44]. No obvious peak at 2.2 Å for Fe–Fe scattering in FeN_5_ SAM was observed, further confirming the absence of iron particles or clusters in the FeN_5_ SAM structure. The coordination environment of N atoms interacting with the single-atom Fe active sites was quantitatively analyzed based on EXAFS best-fitting results in *R* space (Fig. 2c and Supplementary Table S1). The average coordination number of Fe around N are quantified to be approximately 4.88, indicating that the coordination configuration of Fe sites is Fe–N_5_ in the designed SAM. Additionally, wavelet-transform (WT) analysis was conducted to simultaneously reveal the local coordination environment in both *K* and *R* spaces. The highest WT intensities of the Fe K-edge EXAFS at ~ 6.00 Å^−1^ for FeN_5_ SAM are attributed to the Fe–N bond. X-ray photoelectron spectroscopy (XPS) further evaluated the chemical composition and N configurations quantitatively. The high-resolution N 1 s spectra (Fig. 2e) show peaks around 398.2, 400.9, 401.8, and 404.2 eV [31, 45], corresponding to pyridinic, pyrrolic, graphitic, and oxidized N, respectively, indicating nitrogen doping into the carbon matrix. A spectral valley at 399.6 eV, between the dominant pyridinic and pyrrolic peaks, is attributed to Fe-N_x_ species [46], with a configuration percentage of 13.6% Fig. 2f). This result confirms the presence of Fe-N_x_ atomic moiety in the SAM, aligning well with the XANES results.

The FeN_5_ SAM preserves a well-retained one-dimensional CNT-like structure after FePc coupling and low-temperature calcining processes (Supplementary Figure S3). The high-resolution scanning transmission electron microscopy (STEM) image in Fig. 2g reveals that the FeN_5_ SAM comprises some distorted graphitic carbon, with no metal nanoparticles observed. This structure suggests that the atomic Fe is doped within the carbon matrix [47]. High-angle annular dark-field scanning transmission electron microscopy (HAADF-STEM) was employed to examine the atomic-level structure of FeN_5_ SAM. As depicted in Fig. 2h, isolated bright spots, marked in red circles, indicate the presence of numerous dispersed single-atomic Fe sites within the FeN_5_ SAM. Energy-dispersive X-ray spectroscopy (EDS) was also conducted to assess the chemical composition and dispersity. The elemental mapping images in Fig. 2i demonstrate that C, N, and Fe are uniformly distributed throughout the matrix.

The successful fabrication of sensors was first evaluated by cyclic voltammetry (CV) (Supplementary Figure S4 and Figure S5). After modification with FeN_5_ SAM, the electrode exhibits a lower capacity background current, enhancing the detection limits of the electrochemical sensor. Most importantly, the FeN_5_ SAM-based sensor displays a significantly higher current intensity compared to bare LEG and CNT-modified electrodes under the same concentration of UA (Supplementary Figure S6). These results illustrate the improved detection performance achieved by using single-atom materials while benefiting from the 100% utilization of active sites [33, 48, 49]. Figure 3 presents extensive studies on the analytical performance of the sensor for orthogonal UA sensing. The current peak of direct oxidation at the UA sensor is around 0.3 V from the DPV scan. The ratio of Nafion used is optimized first. Different concentrations of Nafion solution mixed with 0.2 mg/mL FeN_5_ SAM were drop-casted onto WE 1. Excessive Nafion affects the rate of mass transfer and reduces sensitivity, while insufficient amounts impact the stability and loading capacity of FeN_5_ SAM. The intensities of the signal response increased rapidly as Nafion concentration decreased and then diminished at lower ratios (Supplementary Figure S7). The maximum DPV current response of WE 1 is achieved at a 0.05% concentration of Nafion. Therefore, a 0.05% concentration of Nafion binder was selected as the optimum condition for further UA sensing. Figure 3a presents the response calibration (after linear baseline current subtraction) over the range of 10 mM to 500 mM. The current intensities of the oxidation peaks increased with increasing concentrations of UA. The corresponding calibration plot is shown in Fig. 3b, with a good linear dependence on the UA concentrations over the entire range. The linear regression equation can be calculated as I (mA) = 101.55 Log (C(mM)) −107.16 with R^2^ = 0.983. The limit of detection (LOD) was calculated as 2.86 mM (S/N = 3). Figure 3c depicts the reproducibility of the DPV response of WE 1 to 300 mM uric acid, measured continuously ten times. Minimal current intensity changes are found (Supplementary Figure S8 and S9a), illustrating that WE 1 has excellent stability for multiple detections. Figure 3d and Supplementary Figure S9b demonstrate the repeatability of the direct oxidation sensor with alternating additions at each specified UA concentration. The results reveal excellent reversibility of the UA oxidation current and highly consistent responses with solution replacement. Figure 3e and Supplementary Figure S9c also show the dynamic calibration of WE 1 across UA concentrations ranging from 50 μM to 500 μM. The consistent sensitivity during both UA increments and decrements indicates a strong potential for reliable operation in field conditions, with minimal response degradation. This repeatability suggests a promising platform for wearable sweat sensing. The sensing selectivity and specificity were further analyzed by evaluating various potential interfering substances that could affect UA detection. These substances include electrolytes and metabolites such as glucose (Glu), K^+^, Na^+^, Cl^−^, lactic acid (LA), and tyrosine (Tyr). As shown in Supplementary Figure S10, there are minimal impacts on the oxidation current signals at around 0.3 V compared to the blank group, suggesting a highly selective UA response. The test solutions contained 300 μM UA with 1 mM electrolyte (NaCl or KCl) or 300 μM interfering metabolites (Glu, LA, or Tyr). As seen in Fig. 3f, the relative responses exhibited a change of less than 5% after the addition of various interfering substances, indicating minimal interference [50, 51]. These results indicate the good specificity of WE 1 towards UA detection.

The FeN_5_ SAM-enzyme cascade electrode (WE 2) operates a biocatalytic sensing platform for UA detection, and its current changes are based on the reduction of intermediates (H_2_O_2_) via chronoamperometry. Several studies have already illustrated that Fe-N_x_-based single-atom materials are able to boost the detection sensitivity towards H_2_O_2_ owing to their 100% active atom utilization and the peroxidase mimicking [31, 52]. Such advantages are expected to enhance the uricase-based biosensing sensitivity of UA levels in human biofluids [39]. The FeN_5_ SAM exhibits the highest reduction current response at −0.37 V (Supplementary Figure S11), which will be applied to CA testing potential conditions. Figure 3g displays chronomamperograms for increasing UA concentrations in 0.1 M PBS. The data indicates that the FeN_5_ SAM-enzyme cascade working electrode (WE 2) is highly sensitive to UA and exhibits a wide linear range, producing well-defined chronoamperograms. The corresponding current response is proportional to the UA concentration, resulting in the linear calibration plot, shown in Fig. 3h. The linear regression equation can be calculated as I (mA) = 3.2711 Log (C(mM)) + 2.6961, with R^2^ = 0.9743. The limit of detection (LOD) is calculated to be 2.73 mM (S/N = 3). The stability of the sensor was evaluated by measuring the response to 5 μM UA for ten times, confirming the feasibility of continuous monitoring. Figure 3i illustrates that the CA current remains highly stable throughout this extended period, with minor overall variations. Similar to the WE1, the WE2 also exhibits good sensing selectivity for the UA detection (Supplementary Figure S12). This wearable UA patch sensor’s good sensing performance is also better or comparable with the performance reported for previous wearable nanomaterial-based UA biosensors (Supplementary Table S2).

Owing to the flexibility of SEBS substrate and the LEGs transferred onto it, the SAM-based UA patch sensor can be comfortably worn on various human body locations, making it suitable for practical applications. As shown in Fig. 4a, the patch is soft and adheres well for long-term wearing on areas prone to perspiration, such as the forehead, neck, or arm. The feasibility of the fabricated SAM-based UA patch sensor was demonstrated by real-time monitoring of sweat UA concentration at these body locations. Figure 4b illustrates that the concentration of UA in sweat can be measured in different areas of the body with minimal variation. Furthermore, we also tested the UA levels of different subjects before and after a purine-rich diet. After fasting overnight, the subjects underwent UA testing through aerobic exercise. The subjects then completed another round of the same UA test 2 h after consuming canned sardines. Figures 4 c–e show the changes in sweat UA of two subjects after overnight fasting and consuming high-purine food. Different subjects exhibited varying signal intensities based on their distinct physiological conditions, but the level of sweat UA significantly increased in all subjects after consuming high-purine foods. Additionally, by comparing the outcomes from consuming 500 g (Fig. 4c) and 250 g (Figs. 4 d–e) of canned sardines in a controlled experiment, we observed that a greater intake of purine-rich diet may result in higher sweat UA levels. The SAM-based UA patch was used to further monitor the dynamic changes in sweat UA levels over a 6-h period before and after consuming a purine-rich diet (Fig. 4f). The UA level in sweat peaks around 2 h after consumption of a purine-rich diet. The same UA concertation trends were observed from WE 1 and WE 2, which further proved that the guaranteed sensing accuracy based on the applied dynamic orthogonal detection approach. Overall, the remarkable ability to monitor sweat UA levels demonstrates the practical applicability of the SAM-based UA patch sensor.

## Conclusion

In summary, the use of single-atom materials has greatly enhanced the performance of a flexible epidermal sensing platform for the orthogonal detection of sweat UA. The integration of a SAM with Fe-N_5_ active sites and LEG-based sensors enhanced sensitivity and expanded the detection range through an innovative orthogonal approach that combines direct oxidation with differential pulse voltammetry and parallel enzyme-cascade biocatalytic amperometric detection. The efficacy of this SAM-based UA patch sensor was confirmed through on-body tests measuring sweat UA levels before and after a purine-rich diet, underscoring its potential for advanced wearable applications in monitoring UA for both biomedical and nutritional purposes. We anticipate that single-atom materials will advance a broad range of wearable sensing applications.

## Supplementary Material

SI

**Supplementary Information** The online version contains supplementary material available at https://doi.org/10.1007/s44258-024-00027-1.

## Figures and Tables

**Fig. 1:**
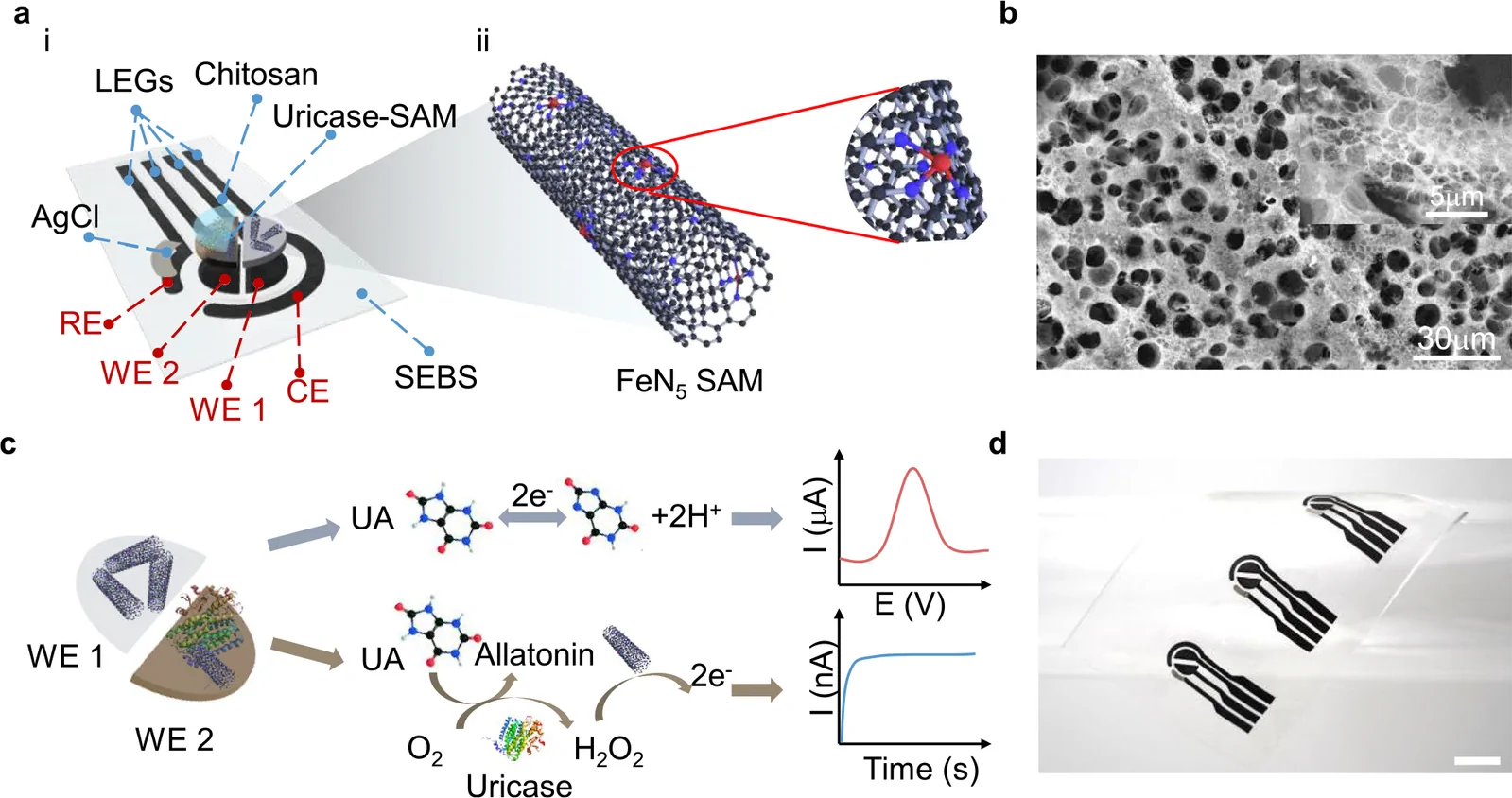
**a** Scheme of the design of SAM-based UA patch sensor with orthogonal detection at WE 1 and WE 2 (i) and the FeN_5_ SAM (ii). **b** Scanning electronic microscopy (SEM) images of LEG. **c** The sensing principle of the orthogonal (anodic and biocatalytic) UA detection. **d** Photo of SAM-based UA patch sensor. Scale bar: 1 cm

**Fig. 2:**
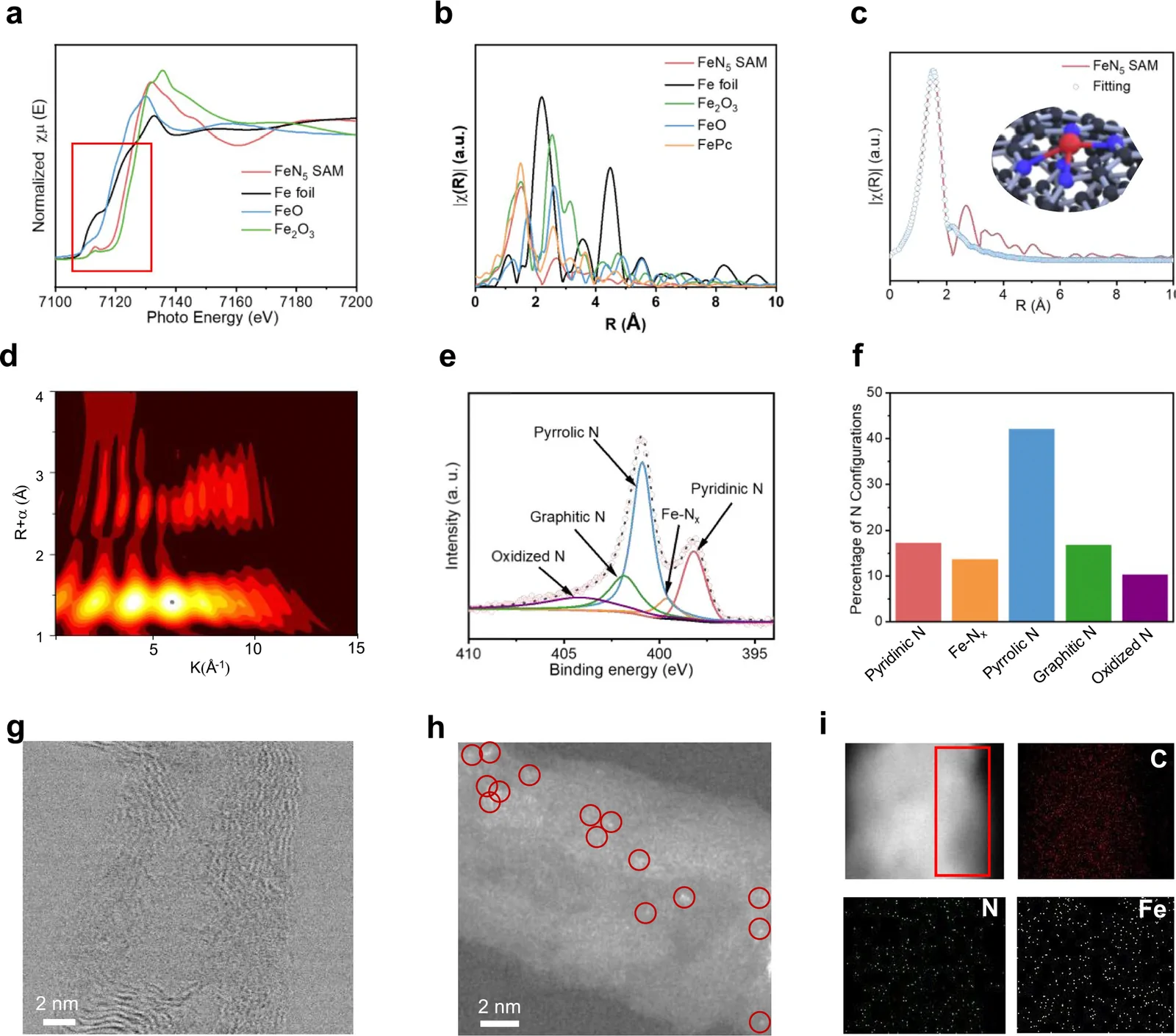
**a** XANES spectra and (**b**) FT-EXAFS spectra of FeN_5_ SAM and reference samples. **c** The corresponding FT-EXAFS fitting curves of the FeN_5_ SAM in R space. **d** WT representation of FeN_5_ SAM. **e** N 1 s XPS spectra of FeN_5_ SAM and (**f**) corresponding percentages of N configurations. **g** STEM-BF image, (**h)** HAADF-STEM image, and (**i**) EDS mapping of FeN_5_ SAM

**Fig. 3:**
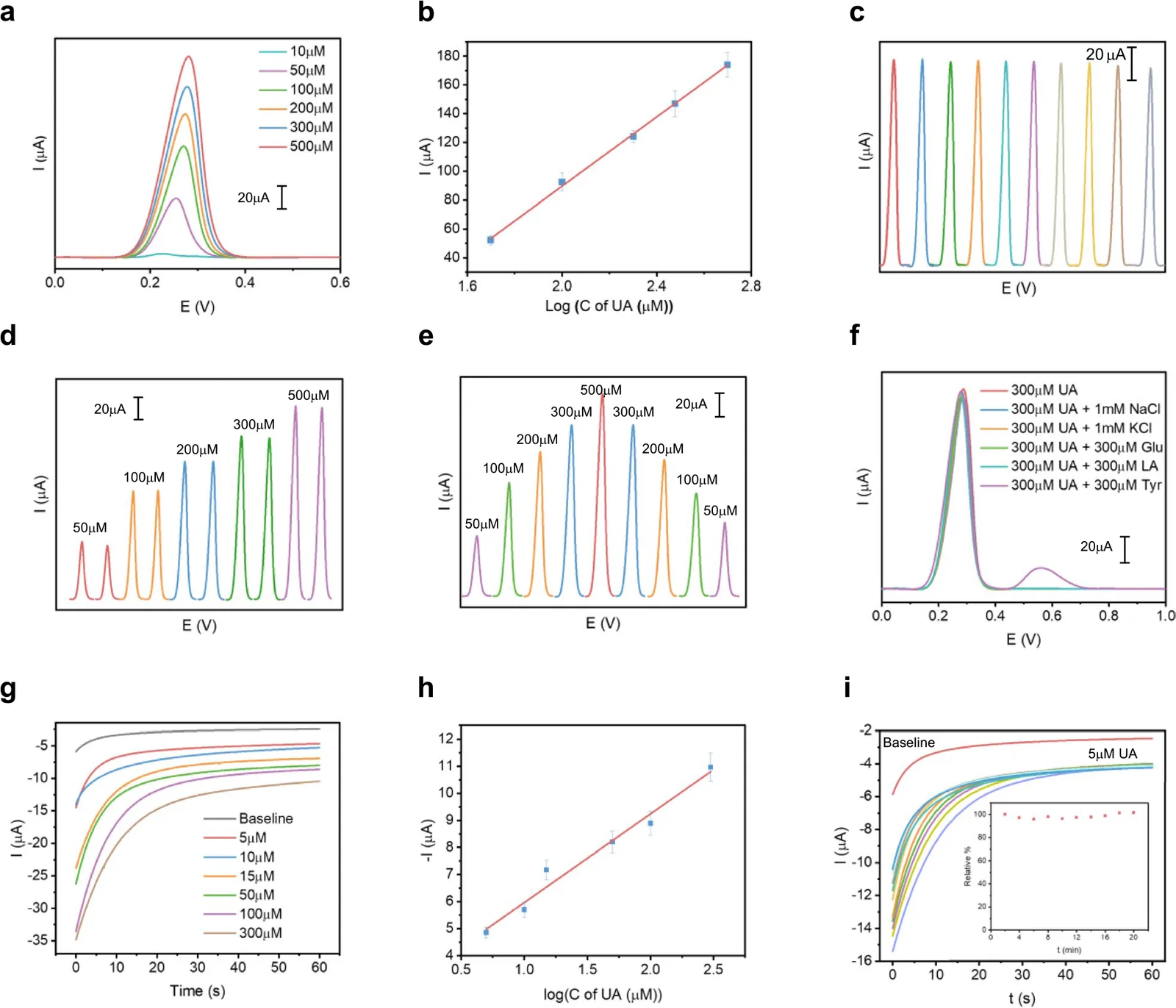
**a** DPV of the WE 1 with different UA concentrations in PBS (**b**) Relationship between peak current and UA concentration in the range of 10–500 mM. **c** Repeatability of the WE 1 sensor, for 10 successive measurements with 300 mM additions every time. **d** Repeatability study of the WE 1 sensor with two alternate additions at each UA concentration, ranging from 50–500 mM. **e** Dynamic increasing and decreasing response calibration study of the WE 1 sensor with different UA additions starting from 50 mM up to 500 mM, and sequential dilutions back to 50 mM. **f** Influence of possible interfering substances on the responses of the WE 1 sensor. **g** Chronoamperograms for increasing UA concentration from 5 mM up to 300 mM. **h** Linear relationship between platform current and UA concentrations. **i** The stability of the electrochemical response to 5 μM UA was assessed ten times

**Fig. 4:**
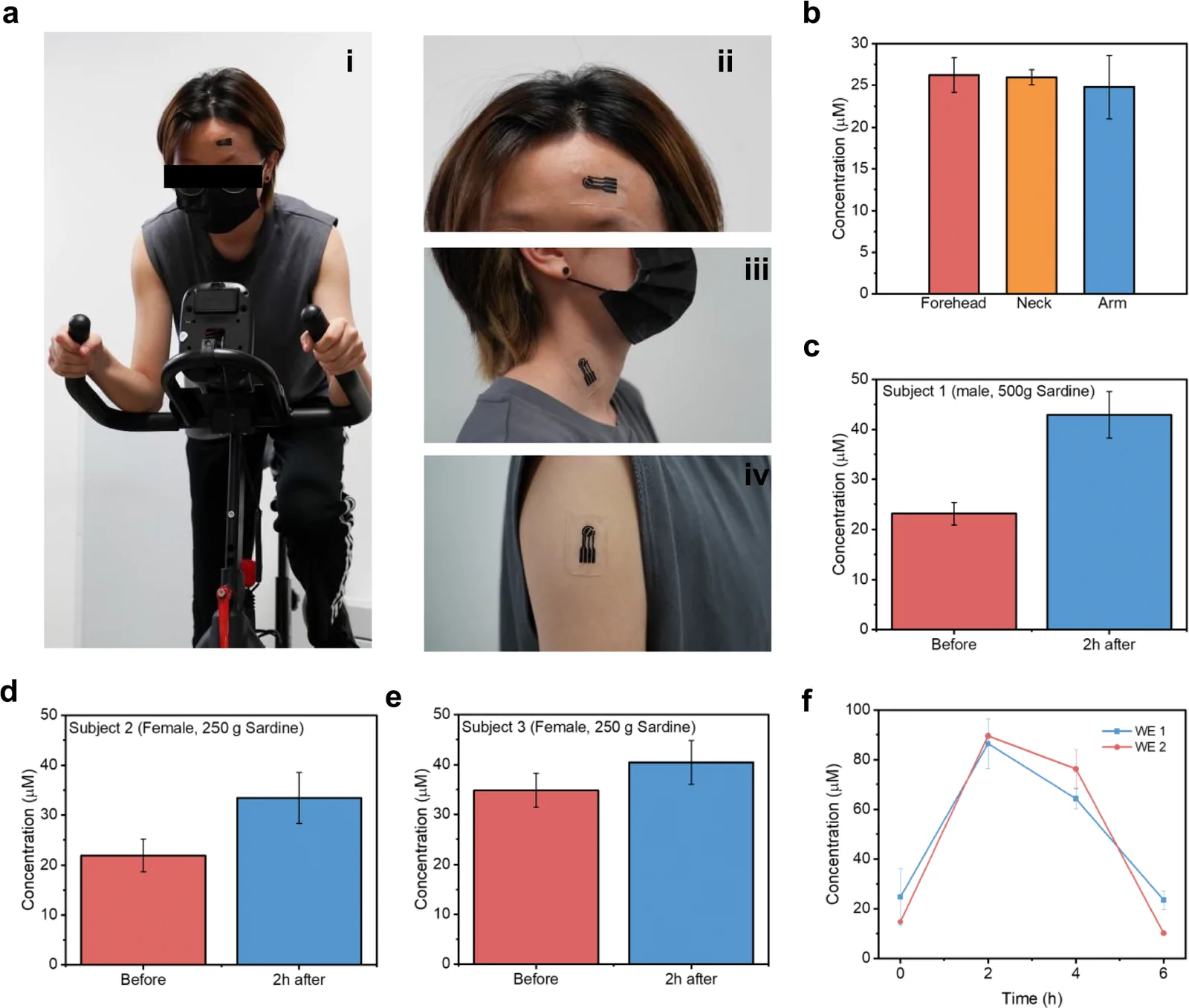
**a** Photographs of a subject wearing the SAM-based UA patch sensor during bicycling (i) and in various locations on the body, including (ii) the forehead, (iii) neck, and (iv) arm. **b** Sweat UA levels monitored on different body locations. **c-e** Sweat UA levels from different subjects before and after a purine-rich diet. **f** Dynamic orthogonal UA concertation changes from one healthy subject before and after a purine-rich diet in a 6-h period

## Data Availability

Data are contained within the article or supplementary material or are available from the authors upon reasonable request.

## Highlight

- A novel single-atom material (SAM) designed with Fe-N_5_ active sites is combined with a laser-engraved graphene electrode to boost highly sensitive uric acid (UA) detection.
- The fabricated SAM-based UA patch sensor exhibits good flexibility and wearability.
- An orthogonal detection approach is utilized, integrating differential pulse voltammetry with biocatalytic amperometric detection, to achieve enhanced accuracy and selectivity in uric acid measurements.
- The SAM-based UA patch sensor has been successfully tested in on-body sweat sensing towards UA, demonstrating its practicality for wearable applications.
