# Supplementary material for: Single-atom materials boosting wearable orthogonal uric acid detection
Source: Med X. Author manuscript; Available in PMC 2026 Oct 24. (PMC13693829; doi:10.1007/s44258-024-00027-1)
Supplement: Supplementary file 1 [file NIHMS2159559-supplement-SI.docx]

**Supplementary information**

Single-atom Materials Boosting Wearable Orthogonal Uric Acid Detection

Shichao Ding,^a,1^ Sitao Li,^b,1^ Zhaoyuan Lyu,^c,1^ Jiachi Zhou,^a^ Selene Tang,^a^ Lingzhe Fang,^d^ Wenjie Zang,^e^ Pengtao Zhang,^a^ Sunjae Kim,^a^ Tao Li,^d,f^ Xiaoqing Pan,^e^ Dan Du,^c^ Yuehe Lin,^c,^* Joseph Wang,^a,^*

^a^ Aiiso Yufeng Li Family Department of Chemical and Nano Engineering, University of California San Diego, La Jolla, CA, 92093 USA

b Shu Chien-Gene Lay Department of Bioengineering, University of California San Diego, La Jolla, CA, 92093 USA

^c^ School of Mechanical and Materials Engineering, Washington State University, PO Box 642920, Pullman, WA 99164, USA

^d^ Department of Chemistry and Biochemistry, Northern Illinois University, DeKalb, Illinois 60115, United States

^e^ Irvine Materials Research Institute (IMRI), Department of Physics and Astronomy, Department of Materials Science and Engineering, University of California Irvine, Irvine, CA, 92697 USA

^f^ X-ray Science Division, Argonne National Laboratory, Lemont, Illinois 60439, United States

^1^ These authors contributed equally

Email: [Josephwang@ucsd.edu](mailto:Josephwang@ucsd.edu); [yuehe.lin@wsu.edu](mailto:yuehe.lin@wsu.edu)

**Characterization**

Transmission electron microscopy (TEM) was employed to examine the morphologies of materials JEM-ARM300F Grand ARM TEM (300 kV) instrument. X-ray photoelectron spectroscopy (XPS) was conducted using a ThermoFisher Thermo with Al Kα radiation to characterize the material. X-ray absorption spectroscopy (XAS) measurements at the Fe K-edge (7,112 eV) were performed at the Advanced Photon Source (APS) on the bending-magnet beamline 12-BM. A Fe metal foil was utilized to calibrate the X-ray beam energy, and it was measured simultaneously with each sample. The radiation was monochromatized using a Si (111) double-crystal monochromator, with harmonic rejection achieved through a harmonic rejection mirror. The FeN_5_ SAM sample spectra were collected in fluorescence mode using a vortex four-element silicon drift detector, while standards (including the foil) were measured in transmission mode. XAS data reduction and analysis were performed using Athena software, and the fitting process was executed using Artemis software within the fitting window between 1-2 Å.

**Electrochemical measurements**

All electrochemical measurements were performed at room temperature using an EmStat3 Blue potentiostat (PalmSens, The Netherlands) controlled by PSTrace software.

**Figure S1** Photos of LEGs (a), after cast with SEBS (b), after transferred on SEBS (c), and after printing AgCl on reference electrode. Scale bars: 1cm.

**Figure S2** Scheme of synthesis of FeN_5_ SAM.

**Figure S3** STEM image of FeN_5_ SAM.

**Figure S4** CV curves of bare LEG and FeN_5_ SAM modified LEG (WE 1)**.**

**Figure S5** CV curves of bare LEG, FeN_5_ SAM modified LEG, and uricase-SAM electrode (WE 2)**.**

**Figure S6** Comparison of DPV sensing ability of FeN_5_ SAM modified LEG, CNT modified LEG, and bare LEG toward 300 μM UA.

**Figure S7** The current intensity from WE 1 toward 300 μM UA modified with different FeN5 SAM Nafion solution ratios**.**

**Figure S8** Normalized oxidation peak current response profile of WE 1.

**Figure S9** The current intensity corresponds to Figure 3c (a), Figure 3b (d) and Figure 3e (c).

**Figure S10** The sensing selectivity of WE1 towards different interfering substances.

**Figure S11** CV curves of WE 2 in 0.1 M PBS and 5mM H_2_O_2_.

**Figure S12** The sensing selectivity of WE2 towards different interfering substances.

**Table S1** Structural parameters of samples obtained by fitting the EXAFS data.

| **Samples** | **Scattering pair** | **CN** | **R(Å)** | **σ^2^(Å^2^)** | **ΔE (eV)** | **R factor** |
| --- | --- | --- | --- | --- | --- | --- |
| **FeN_5_ SAM** | Fe-N | ~4.88 | 2.048 | 0.0159 | -27.35 | 0.00045 |

N: average coordination number; R: path distance, σ^2^: Debye-Waller factor, ∆E: threshold energy correction.

**Table S2** Comparison the materials, method, and performance of SAM-based UA patch sensor with reported wearable UA sensors

| **Materials** | **Techniques** | **Linear range** | **Limits of Detection** | **Ref.** |
| --- | --- | --- | --- | --- |
| **Ni/RGO/CCF** | DPV, CV | 10 - 60 μM | 5.083 μM | [1] |
| **ink-coated thread** | i-t | 0 - 800 μM | N/A | [2] |
| **FeSAs** | DPV | 1 - 425 μM | 0.99 μM | [3] |
| **BDDE** | DPV | 0.5 - 50 μM | N/A | [4] |
| **Boron-Doped Graphene** | DPV | 0 - 50 μM | N/A | [5] |
| **N-rGO/Au DAs** | DPV | 5 - 600 μM | 3.7 μM | [6] |
| **MWCNT-COOH** | DPV | 0 - 40 μM | 3.58 μM | [7] |
| [**uricase@MAF-7**](mailto:uricase@MAF-7) | DPV | 2 -7 μM | 0.34 μM | [8] |
| **FeN_5_ SAM** | DPV | 10 - 500 μM | 2.86 μM | This Work (WE1) |
| **FeN_5_ SAM-uricase** | i-t | 5 - 300 μM | 2.73 μM | This Work (WE2) |

**References**

[1] A. Singh, A. Sharma, S. Arya, Deposition of Ni/RGO nanocomposite on conductive cotton fabric as non-enzymatic wearable electrode for electrochemical sensing of uric acid in sweat, Diamond Relat

Mater, 130(2022).

[2] X.Y. Liu, P.B. Lillehoj, Embroidered electrochemical sensors on gauze for rapid quantification of wound biomarkers, Biosens Bioelectron, 98(2017) 189-94.

[3] Yong Zhang, Changjun Hou, Peng Zhao, Xin Zeng, Yiyi Liu, Jian Chen, Yifan Gao, Cuncun Wang, Jingzhou Hou, and Danqun Huo. ACS Applied Materials & Interfaces 2023 15 (37), 43541-43549

[4] S.Y. Lin, X.B. Cheng, B. Wang, W.Z. Yu, D. Ly, S. Emaminejad, A Fouling-Resistant Voltametric Sensing System for Wearable Electroactive Biomarker Monitoring, Journal of Microelectromechanical S14 Systems, 29(2020) 1059-63.

[5] Y.X. Wang, M. Rinawati, J.D. Zhan, K.Y. Lin, C.J. Huang, K.J. Chen, et al., Boron-Doped Graphene Quantum Dots Anchored to Carbon Nanotubes as Noble Metal-Free Electrocatalysts of Uric Acid for a Wearable Sweat Sensor, Acs Applied Nano Materials, 5(2022) 11100-10.

[6] Yao Chen, Guanglei Li, Wenjing Mu, Xinhao Wan, Danfeng Lu, Jie Gao, and Dan Wen, Analytical Chemistry 2023 95 (7), 3864-3872

[7] Li Z, Wang Y, Fan Z, Sun Y, Sun Y, Yang Y, Zhang Y, Ma J, Wang Z, Zhu Z. A Dual-Function Wearable Electrochemical Sensor for Uric Acid and Glucose Sensing in Sweat. Biosensors (Basel). 2023 Jan 6;13(1):105.

[8] Jingyu Xiao, Yong Luo, Lei Su, Jiafa Lu, Wei Han, Tailin Xu, Xueji Zhang, Hydrophilic metal-organic frameworks integrated uricase for wearable detection of sweat uric acid, Analytica Chimica Acta, Volume 1208, 2022.
